# Electronic system with memristive synapses for pattern recognition

**DOI:** 10.1038/srep10123

**Published:** 2015-05-05

**Authors:** Sangsu Park, Myonglae Chu, Jongin Kim, Jinwoo Noh, Moongu Jeon, Byoung Hun Lee, Hyunsang Hwang, Boreom Lee, Byung-geun Lee

**Affiliations:** 1 Department of Nanobio Materials and Electronics, Gwangju Institute of Science and Technology, Gwangju, Korea 500-712; 2 Department of Mechatronics, Gwangju Institute of Science and Technology, Gwangju, Korea 500-712; 3 Department of Medical System Engineering, Gwangju Institute of Science and Technology Gwangju, Korea 500-712; 4 School of Information and Communications, Gwangju Institute of Science and Technology, Gwangju, Korea 500-712; 5 Department of Materials Science and Engineering, Pohang University of Science and Technology, Pohang, Korea 790-784

## Abstract

Memristive synapses, the most promising passive devices for synaptic interconnections in artificial neural networks, are the driving force behind recent research on hardware neural networks. Despite significant efforts to utilize memristive synapses, progress to date has only shown the possibility of building a neural network system that can classify simple image patterns. In this article, we report a high-density cross-point memristive synapse array with improved synaptic characteristics. The proposed PCMO-based memristive synapse exhibits the necessary gradual and symmetrical conductance changes, and has been successfully adapted to a neural network system. The system learns, and later recognizes, the human thought pattern corresponding to three vowels, i.e. /a /, /i /, and /u/, using electroencephalography signals generated while a subject imagines speaking vowels. Our successful demonstration of a neural network system for EEG pattern recognition is likely to intrigue many researchers and stimulate a new research direction.

Artificial neural networks (ANNs) that utilize parallel computing are known to be an effective method of processing large datasets, such as for pattern recognition, classification, and clustering. In such fields, software-realized ANNs have already been developed for commercial use, but their operating speed is generally insufficient for increasingly complex networks. The alternative approach of a hardware neural network (HNN) has been studied for some time, with analogue and digital techniques that implement artificial neurons and synapses^1^,^2^,^3^,^4^,^5^,^6^ having a clear speed advantage over software-based ANNs^7^,^8^,^9^. However, synaptic devices that use analogue circuits require too much power, whereas digital approaches use too many transistors in a single synaptic device. Hence, their current functionality limits the ability of HNNs to replace their software counterparts. The problem of integrating a synaptic device into a small chip without burning too much power is one of the major obstacles in developing advanced HNNs. To enable their use in HNNs, synaptic devices must satisfy two conditions. First, they should have a simple, two-terminal architecture, allowing high integration density to be achieved via a cross-point array. Second, the weight of the devices should change gradually with the bias voltage, and the rate at which the weight increases and decreases should be symmetric.

Ever since the concept of memristive synapses were introduced in the late 1960s, they have been considered as promising candidates for synaptic devices because of their simplicity and functional similarity to a synapse. Recent advances in nanoscale metal-insulator-metal devices that have memristive characteristics such as phase-change memory (PRAM)^10^, conductive-bridge memory (CBRAM)^11^, and oxide based memory (RRAM)^12^ have strengthened this belief. However, the conducting filament formation of current memristive synapse causes significant variations in resistance, preventing the desired gradual and symmetric change in conductance. Although significant efforts have been made to develop HNNs using such devices, the memristive HNNs reported to date only show the possibility of system learning using a simple crossbar array^13^. In this paper, we present a high-density cross-point memristive synapse array with improved synaptic characteristics, and describe a learning scheme to mitigate the unintended switching problem often encountered with cross-point arrays. The conductance of our memristive synapse changes more gradually and symmetrically in the presence of voltage pulses above a certain threshold voltage; otherwise, the memristive synapse retains its conductance.

We also present experimental results from a memristive HNN system that recognizes the human thought pattern relating to three vowels, /a/, /i/, and /u/, based on electroencephalography (EEG) signals generated while a subject imagines speaking these vowels. We believe that this is the first primitive prototype of an electronic system that utilizes a cross-point memristive synapse array for EEG pattern recognition. Our results suggest a new research direction for memristive HNNs.

## Results

### System description

**Figure 1:**
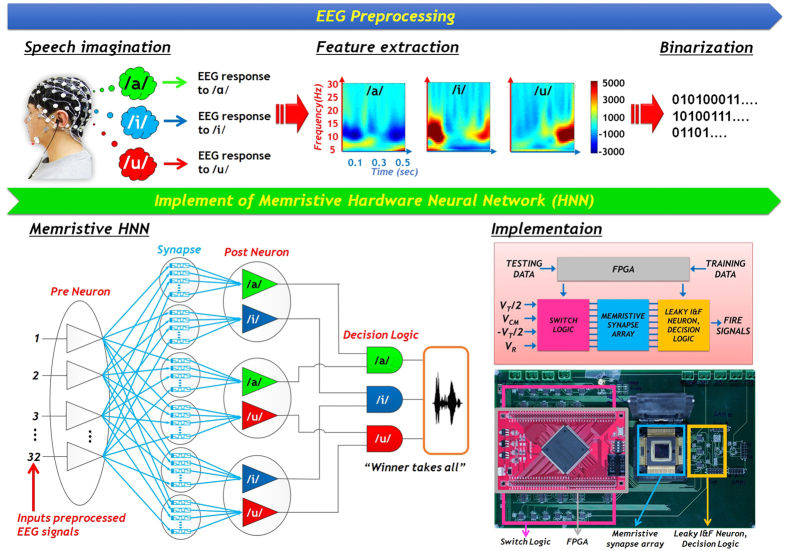
Proposed memristive HNN system for EEG pattern recognition Schematic illustrations and images of components for a proposed electronic system with memristive synapse. It can be categorized by two approaches: EEG preprocessing and implement of memristive hardware neural network.

As shown in fig. 1, the proposed system can be divided into two functional blocks. The first block, mainly realized in software, captures the EEG signals from a subject, and processes them to extract the distinct features of three vowels. Each feature is converted into a series of 32-bit binary code to be used by the 32 pre-neurons to generate a spike signal. The second block is a single-layer neural network that consists of 32 pre-neurons, 192 memristive synapses, and 6 post-neurons. The pre-neurons are hard-coded into a field-programmable gate array (FPGA), and 192 memristive synapses in a cross-point memristive synapse array are selected for synaptic interconnection. A leaky integrate-and-fire neuron is used as the post-neuron, and decision logic determines which post-neuron fires first based on the output signals from the post-neurons. The system’s overall control signals are also generated by the FPGA. Note that, to increase the system’s recognition rate, a pairwise comparison is performed. In other words, the post-neurons are paired into three groups, and each group compares two of the three vowels, i.e. /a/ vs /i/, /a/ vs /u/, and /i/ vs /u/.

The system operates in two different modes, learning and testing. In learning mode, the system’s learning network adjusts the conductance of memristive synapses using spike signals generated according to the EEG signals of the pre-selected vowel. In testing mode, the subject randomly chooses and imagines saying one of the three vowels. The system analyses the subject’s EEG signal, and attempts to determine which of the three vowels was chosen.

### EEG analysis of imagined speech

**Figure 2:**
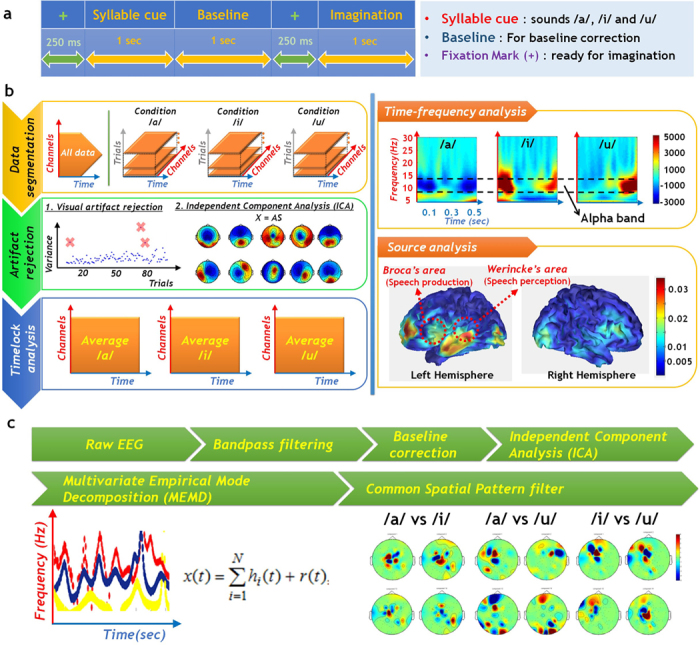
EEG analysis and processing (**a**) Experimental paradigm for EEG study consists of four parts. (**b**) EEG data analysis. (**c**) Signal processing.

The experimental paradigm for this study is described in fig. 2(a). The acquired EEG data are segmented according to the trial and the stimuli associated with /a/, /i/, and /u/ (Fig. 2(b), top left). The segmented data are then analysed to identify the distinct features of the three experimental conditions (/a/, /i/, and /u/).

Initially, continuous raw EEG data are segmented into each condition, with artifact rejection used to counter the low signal-to-noise ratio of EEG data. To estimate the activity evoked by the speech imagery data, we averaged the EEG data over all trials for each condition, a process known as time-lock analysis. A time-frequency analysis was then conducted using the Morlet wavelet. As shown in the top right panel of fig. 2(b), the alpha band (8–12 Hz) activities of each vowel are distinct. To identify the current sources of the speech imagery EEG data, we conducted source localization using a Laplacian-weighted current density estimator. As shown on the right of the second row in fig. 2(b), the current sources of speech imagery EEG data were located close to Broca’s and Wernicke’s areas, as well as the primary and secondary cortex. Broca’s area and Wernicke’s area are closely related to speech production and perception, respectively.

Based on these results, we extracted the features needed to classify speech imagery EEG data for each vowel. First, we applied an IIR band-pass filter (Butterworth order: 5, bandwidth: 8–30 Hz) and baseline correction using pre-stimulus data to eliminate residual noise. We then used independent component analysis to eliminate artefacts, and decomposed the EEG data into intrinsic mode functions (IMFs) using multivariate empirical mode decomposition (MEMD). After MEMD, the dominant alpha-band IMF was extracted based on the time-frequency analysis. To enhance the classification performance, we applied a common spatial pattern filter that maximizes the variance between groups. Finally, we binarized the extracted features for input to the hardware.

### Considerations for implementing a cross-point memristive synapse array

**Figure 3:**
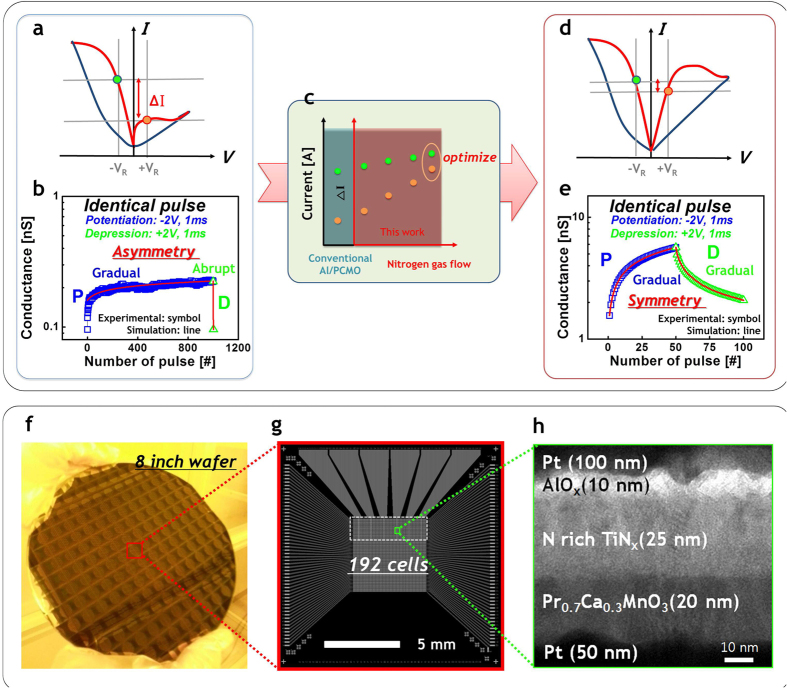
Considerations for a memristive synapse implementation in cross-point array (**a**) The asymmetric I-V characteristics of Al/PCMO structure. (**b**) The asymmetric conductance response of Al/PCMO memristive synapse in identical pulse scheme. (**c**) By optimizing nitrogen concentration during TiN_x_ deposition. (**d**) Symmetric I-V characteristics of AlO_x_/TiN_x_/PCMO structure. (**e**) The symmetric conductance response of AlOx/TiNx/PCMO memristive synapse in identical pulse scheme. (**f**) A Photograph of a memristive synapse array in 8 inch wafer. (**g**) A SEM view image of the cross-point memristive synapse array. (**h**) A TEM view image of the cross-point memristive synapse array.

To simplify the architecture of HNN, memristive synapses with advanced synaptic behavior should use identical pulses and a simple cross-array structure with two terminals. Advanced synaptic behavior implies not only gradual, but also symmetric responses in both potentiation and depression. The asymmetric *I-V* characteristics (|current (@ + 1 V)|<<|current (@ − 1 V)|) of a previous Al/PCMO memristive synapse^14^ are shown in fig. 3(a). The Al/PCMO memristive synapse has an inhomogeneous barrier at the interface between the Al (reactive metal) and PCMO (p-type semiconductor)^15^. Because the conductance of the memristive synapse must be simultaneously updated in the HNN, the asymmetric conductance response (Fig. 3(b)) from identical pulses limits the overall accuracy of recognition^16^. Advanced synaptic behavior also requires the conductance to vary continuously when a relatively large voltage bias is applied, but the conductance should remain constant when a smaller or no bias is applied. This characteristic is vital for the nondestructive read/write scheme that eliminates the unintended switching issue, as explained in detail below. A simple, two-terminal cross array structure is necessary for the practical implementation of high-density HNN^17^.

In this work, we have enhanced the synaptic behavior without a complicated programming scheme by engineering a PCMO-based memristive synapse. We optimized the nitrogen concentration during TiN_x_ deposition (Fig. 3(c)) to minimize the inhomogeneous barrier between the top electrode(TE)–PCMO interface^18^. This allowed us to obtain symmetric responses for the I-V and conductance characteristics (Fig. 3(d)). We also developed a 200 mm wafer-scale PCMO-based memristive synapse (Fig. 3(f)) that exhibits excellent switching uniformity and analogue memory behaviour (see Supplementary Fig. S1 online). Figure 3(g) shows a typical scanning electron microscope (SEM) image of a 32 × 6 array (192 cells) of the proposed PCMO-based memristive synapse. (Although memristive synapse arrays can be fabricated up to a size of 11 kbit, our memristive HNN allows only 32 × 6 arrays because of the limited switch logic.) If the memristive synapse and CMOS circuits are integrated in a single chip, there is no critical need for high-density memristive HNN. Figure 3(h) shows a TEM image of our PCMO-based memristive synapse fabricated using the 200 mm wafer process. It consists of active Pt/AlO_x_/TiN_x_/Pr_0.7_Ca_0.3_MnO_3_/Pt (from top to bottom) devices (see methods part for details).

### Memristive HNN learning

To classify feature code by using memristive HNN, we require a learning algorithm. We propose a modified learning algorithm based on a conventional and widely used supervised learning algorithm^19^. In conventional supervised learning, feature codes and label data are required. The firing neuron is predetermined by the label data and synaptic weights are updated by feature codes, allowing the predetermined neuron to fire. To apply this algorithm to memristive HNN, the unintended switching problem must be carefully handled.

In the learning mode(rather than testing mode), the unintended switching issue causes non-linear conductance changes of unwanted memristive synapses. Generally, in memory applications^20^, this problem is easily solved using the half-bias scheme. However, it is not so simple in memristive HNN, because multiple memristive synapses must be updated at the same time (see Supplementary Fig. S2 online).

**Figure 4:**
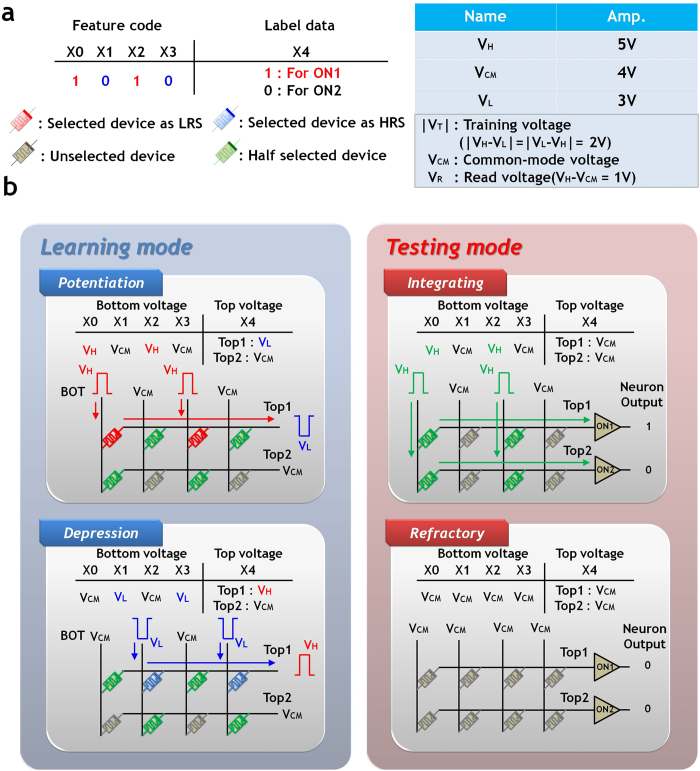
The proposed learning scheme (**a**) An example of the feature code and label data to train memristive synapses of top1. (**b**) A schematic of the 4 pre-neurons and 2 post-neurons HNN according to operation phase.

**Figure 5:**
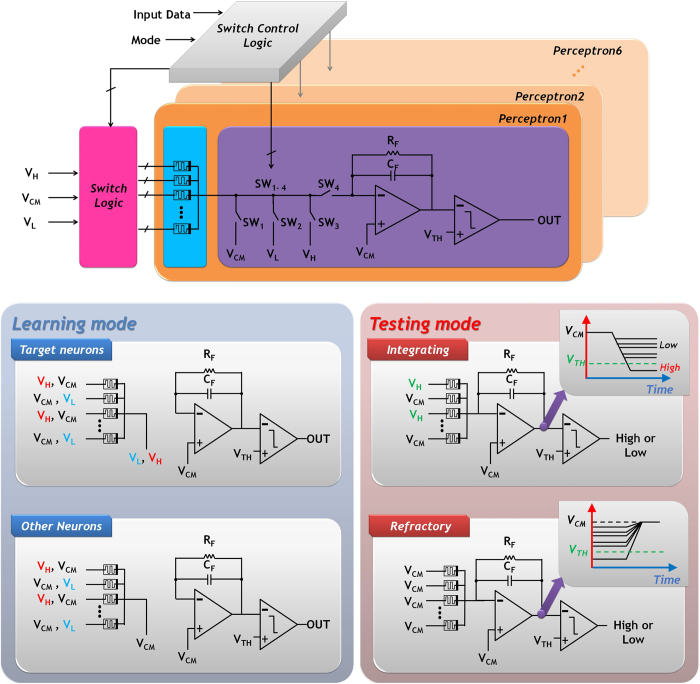
Circuit Implementation The circuit includes a switch array, switch control logic, 32x6 cross-point memristive synapse array and six neuron circuits. Each neuron contains OPAMP based inverting integrator and comparator. Also, a schematic of HNN circuit according to operation phase are shown in this figure.

To overcome this problem, the conventional half-bias scheme was modified to enable its application to memristive HNN. The proposed learning requires two operation phases for a given feature code, potentiation and depression. The spike signals applied to the top electrode (TE) and bottom electrode (BE) are determined according to label data and feature codes. Note that when the pulse amplitude between the TE and BE is in the range |V|<V_R_  ≈ 1 V (where V_R_ is the read voltage), the state changes are negligible. To update the memristive synapse conductance, the training voltage (|V_T_| > V_R_) should be supplied to the target cell by applying V_H_ (or V_L_) and V_L_ (or V_H_) to the TE and BE of the cross-point array, respectively. As shown in fig. 4, the proposed learning algorithm for a single feature code can be easily explained with simple memristive HNN (4 pre-neurons & 2 post-neurons) and detail of the circuit implementation are presented in the bottom-left of fig. 5.

In the potentiation phase, if the label data is ‘1’, the TE of all synapses in the first row (labelled as Top1) is connected to V_L_, then the BE of each synapse is either connected to V_H_ for a feature code of ‘1’, or to V_CM_ for ‘0’. As V_H_ and V_L_ are 5 V and 3 V spike signals, respectively, the synapse whose BE is connected to V_H_ sees a 2 V potential difference across it and increases its conductance. Whereas the synapses in the first row are trained, those in the second row retain their states by applying V_CM_ to Top2. This guarantees that the potential difference across the synapses in the second row does not exceed V_R_ and minimizes unintended switching effect.

In the depression phase, conductance of the selected synapses reduces by applying V_H_ and V_L_ to the TE and BE of the associated synapses, respectively. This process is repeated until all the memristive synapses are trained for all feature codes.

Notice that the initial conductance was set to a mid-value between minimum (≈1.5 nA/V) and maximum (≈5.5 nA/V) conductance. As shown in fig. 3(e), the change in conductance for each learning pulse depends on the current state of a memristive synapse. When a memristive synapse is in its low-conductance state, pulses will result in a rapid increase in conductance. The change rate is about 0.2 ~ 0.5 nA/V for each pulse. The change rate gradually decreases to about 0.02 ~ 0.05 nA/V as the synapse approaches its high-conductance state. Finally, the conductance becomes saturated.

### Memristive HNN testing and classification results

In testing mode, applied feature codes to the memristive HNN are recognized by the decision logics based on the output signals of the post-neurons. Like the learning mode, the testing mode requires two operation phases, integrating and refractory. The spike signals applied in these periods are shown in the bottom-right of fig. 4(b) and detail of circuit implementation is shown in the bottom-right of fig. 5.

A leaky integrate-and-fire neuron, including a comparator and inverting leaky integrator, is used as the post neuron^21^. Integrator output decreases as the current flowing through the memristive synapse accumulates on the integration capacitor during the integrating phase. If the trained conductance is large, we observe a large amplitude in the input current. Thus, the output voltage quickly decreases. As soon as the integrator output drops below a neuron’s threshold voltage (V_TH_ = 3 V), the comparator output generates a high value (logic value of 1), and the neuron is assumed to have fired. Note that the time required for the integrators to reach V_TH_ with a given feature code depends on the trained conductance of the memristive synapse. After the integrating phase has completed, the refractory period starts. During this phase, the charge on the integration capacitor needs to be fully discharged through the leaky path to prepare the neuron for the next feature code.

**Figure 6:**
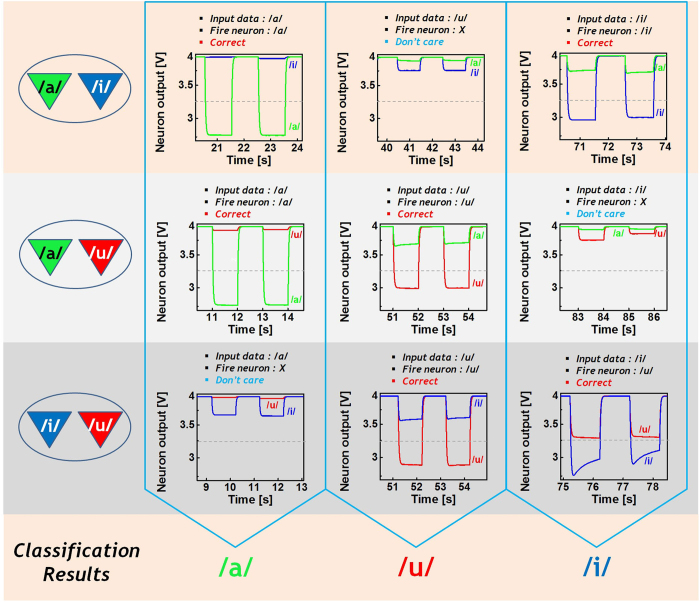
The recognition results of speech imagination Measured output of each integrator during testing mode is shown. When feature code of /a/ is used as an input of memristive HNN, its results is shown in first column. In the cases of /u/ and /i/, its results are also shown in second and third column, respectively.

The output measured at each integrator during the testing mode is shown in fig. 6. The outputs from six integrators for the feature /a/ are shown in the first column. As expected, the integrators’ output drops almost linearly to their saturation value which depends on the conductance of the memristive synapse and the RC time constant of the integrator, at different rates after an initial reset (=4 V). Thus, for /a/, two of the six neurons generate a fire signal as the integrators’ output reaches V_TH_ and the feature code applied to the system is finally recognized as /a/ through the decision logic. After the integration phase, all integrators’ outputs are reset to 4 V during the refractory phase. The output responses for /u/ and /i/ are also shown in fig. 6, which are similar results as for /a/.

## Discussion

For the first time, we have developed an electronic system implemented as a memristive HNN for EEG pattern recognition. We engineered cross-point memristive synapse array using a 200 mm wafer scale. Based on the EEG results, we extracted the features for classifying speech imagery EEG data for each vowel. We tested the proposed memristive HNN system using preprocessed EEG feature codes, and were able to achieve impressive classification results (see Supplementary Fig. S3 online). This result provides a pathway for the future design of high density memristive HNN by overcoming the scalability, connectivity, and synaptic density challenges.

## Methods

### EEG experiment

This study was approved by the Institutional Review Board (IRB) of Gwangju Institute of Science and Technology (GIST) and all subjects provided written informed consent. The experiments were carried out in accordance with the approved guidelines of IRB. Six healthy subjects participated in this study. None of the subjects have experienced any neurological disorders, and all are right-handed. A pre-test familiarization experiment was conducted for all subjects. EEG data were recorded at 250 samples/s using a 64-channel EEG device made by Electrical Geodesics, Inc. Electrodes were placed according to the international 10-20 system, and those corresponding to the sensory motor cortex (based on neurophysiological insight) were used for further analysis. We recorded EEG data in a dimly lit room. All experiments were performed in complete silence to reduce the occurrence of noise. During the EEG recording, subjects were seated in a comfortable armchair wearing a set of earphones (ER-4P, Etymotic Research). The experiments consisted of four parts, and the total length of each trial was 3.5 s. The first part, lasting 1 s, was the syllable cue period, in which an /a/, /i/, or /u/ sound was provided at random to the subjects through the earphones. The EEG data recorded during the next period was used for baseline correction. In the last part, subjects imagined speaking the vowel, /a/, /i/, or /u/, which they had heard during the syllable cue period. A fixation mark was presented on the computer monitor before every syllable cue, and an imagination period lasting 250 ms allowed the subjects to prepare to listen or imagine the syllable.

We conducted 100 trials for each syllable; thus, a total of 300 trials per subject were recorded. The IIR band-pass filter was applied to the raw EEG data to eliminate residual artefacts during the analysis period (Butterworth order: 5, bandwidth: 8–30 Hz). The baseline correction was conducted using EEG data recorded during the baseline correction period. MEMD was used to extract the task-related time-frequency components from the filtered EEG data. To extract more effective features, we used the common spatial pattern that maximized the variance between the groups (/a/, /i/, and /u/). The feature vectors extracted from the common spatial pattern were binarized to 32 bits. The preprocessed 32-bit training patterns are shown in Supplementary Table. S1-3 online. The total number of different training and testing patterns are 65 and 15, respectively.

### Structure and fabrication of memristive synapses

The cross-point memristive synapse array consists of active Pt/AlO_x_/TiN_x_/Pr_0.7_Ca_0.3_MnO_3_/Pt (from top to bottom) devices with various cell numbers (from 192 to 11664 cells). Supplementary Fig. S4 online illustrates the cross-point region between the two electrodes. To fabricate this structure, a bottom electrode consisting of a 50 nm-thick Pt layer (deposited by electron beam evaporation) and a 20 nm-thick polycrystalline PCMO film (deposited by RF magnetron sputtering) were applied by conventional lithography and reactive ion etching. During the PCMO deposition, the substrate temperature was maintained at 600 °C. Subsequently, an 80 nm-thick SiN_x_ layer was deposited by plasma enhanced chemical vapour deposition, followed by the formation of holes by conventional lithography and reactive ion etching. For the top electrode, 25 nm-thick TiNx (N_2_ ambient), 10 nm-thick AlO_x_ layer (as an internal resistor), and 80 nm-thick Pt were sequentially deposited and patterned.

### HNN circuit implementation and measurement

Supplementary Fig. S5 online shows the single-layer HNN implemented on printing circuit board. The circuit includes a switch array, switch control logic, 32 × 6 cross-point memristive synapse array, and six neuron circuits. The cross-point memristive synapse array is fabricated in 200 mm wafer scale, and the switch control logic, which selects an appropriate spike signals for each operation mode based on the feature codes, is implemented in a field-programmable gate array. The others are implemented with commercial chip components, such as OPAMP and switch circuits. Also, integrators output are captured by NI data acquisition device (USB-6281). The average power consumption of the memristive HNN system is 47.9 mW for each matching operation, and the input current of each perceptron is about 90 nA.

## Author Contributions

S. P., M. C., B. L. and B-G. L. conceived the idea, designed the experiments and interpreted the results; J. K. performed the EEG experimental work; J. N performed the simulations; M. C performed the hardware implementation; H. H. and B-G. L. planned and supervised the project; M. J., B. L. and B. H. L. contributed to discussion throughout the project; S. P and M. C., co-first authors, wrote the manuscript; M. C. and B.-G. Lee revised the manuscript.

## Additional Information

**How to cite this article**: Park, S. *et al*. Electronic system with memristive synapses for pattern recognition. *Sci. Rep.* **5**, 10123; doi: 10.1038/srep10123 (2015).

## Supplementary Material

Supplementary Information
