# Supplementary material for: Electronic system with memristive synapses for pattern recognition
Source: Sci Rep. 2015 May 5;5:10123. doi: 10.1038/srep10123 (PMC4419523; doi:10.1038/srep10123)
Supplement: Supplementary Information [file srep10123-s1.doc]

**Supplementary Information**

**Electronic system with memristive synapses for pattern recognition**

Sangsu Park^1†^, Myonglae Chu^2†^, Jongin Kim^3^, Jinwoo Noh^1^, Moongu Jeon^4^, Byoung Hun Lee^1^, Hyunsang Hwang^5^, Boreom Lee^3*^, and Byung-geun Lee^2*^

^1^Department of Nanobio Materials and Electronics, Gwangju Institute of Science and Technology, Gwangju, Korea 500-712.

^2^Department of Mechatronics, Gwangju Institute of Science and Technology, Gwangju, Korea 500-712.

^3^Department of Medical System Engineering, Gwangju Institute of Science and Technology Gwangju, Korea 500-712.

^4^School of Information and Communications, Gwangju Institute of Science and Technology, Gwangju, Korea 500-712.

^5^Department of Materials Science and Engineering, Pohang University of Science and Technology, Pohang, Korea 790-784.

^†^These authors contributed equally to this work(co-first author).

*Correspondence and requests for materials should be addressed to Boreom Lee([**leebr@gist.ac.kr**](mailto:leebr@gist.ac.kr)) or Byung-geun Lee ([**bglee@gist.ac.kr**](mailto:bglee@gist.ac.kr)),

**
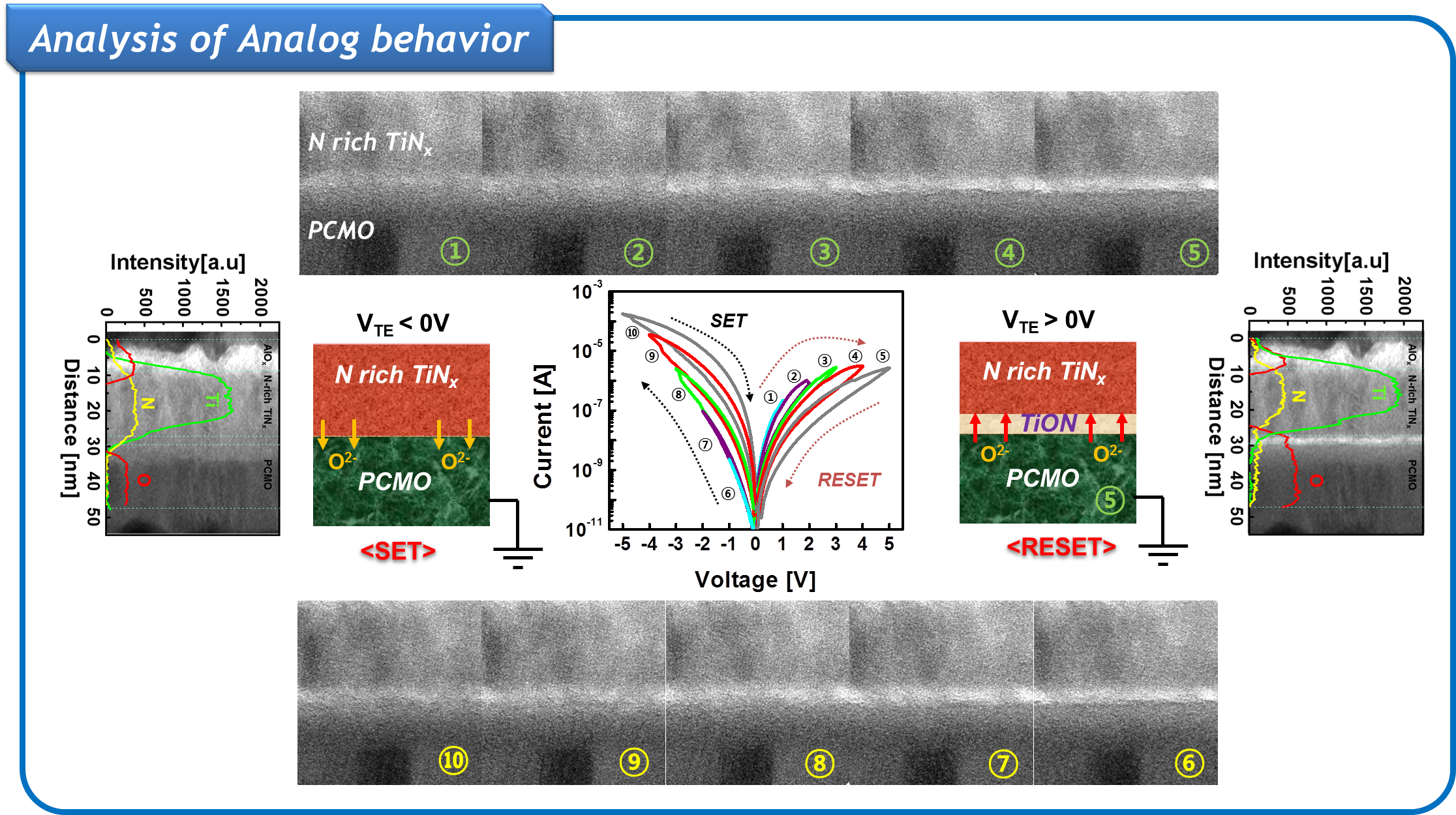
**

**Supplementary Figure S1 ǀ Analysis of analog behavior.** By using in-situ TEM analysis, we obtained the real-time TEM images. This result shows the analog memory characteristics in dc mode by using repeated incremental negative and positive voltage sweeps. The difference of residual oxide layer between LRS and HRS was shown in the sequential steps. A bright layer is observed at interface between TiN_x_ and PCMO. Oxygen elements are detected at interface between TiN_x_ and PCMO in HRS.


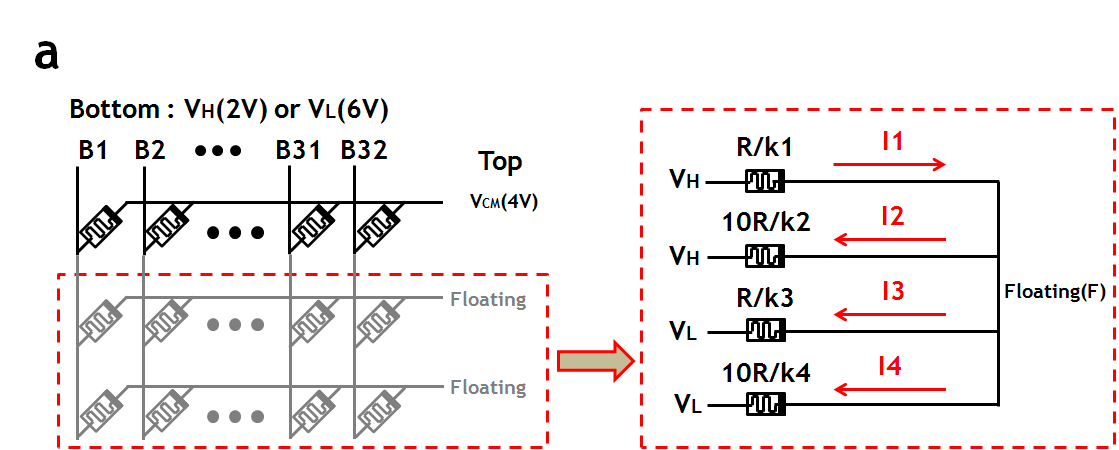


where

$\begin{matrix} V_{H} = Learning voltage for HRS, V_{L} = Learning voltage for LRS \\ LRS value=R, HRS value = 10\cdot R \\ N= \# of memristivedevices \\ k1+k3= \#of LRS=N-k \\ k2+k4= \#of HRS =k \end{matrix}$ $\begin{matrix} I_{1}=I_{2}+I_{3}+I_{4} \\ \to\frac{k1\cdot(V_{H}-F)}{R}=\frac{k2\cdot(F-V_{H})}{10\cdot R}+\frac{k3\cdot(F-V_{L})}{R}+\frac{k4\cdot(F-V_{L})}{10\cdot R} \\ \to10\cdot k1\cdot(V_{H}-F) = k2\cdot(F-V_{H})+10\cdot k3\cdot(F-V_{L})+k4\cdot(F-V_{L}) \\ \to F\cdot(10\cdot k1+k2+10\cdot k3+k4) = 10\cdot k1\cdot V_{H}+k2\cdot V_{H}+10\cdot k3\cdot V_{L}+k4\cdot V_{L} \\ F=\frac{V_{H}\cdot(10\cdot k1+k2)+V_{L}\cdot(10\cdot k3+k2)}{10\cdot(k1+k3)+k2+k4}=\frac{V_{H}\cdot(10\cdot k1+k2)+V_{L}\cdot(10\cdot k3+k2)}{10\cdot N-9\cdot k} \end{matrix}$When N is 32 for this work


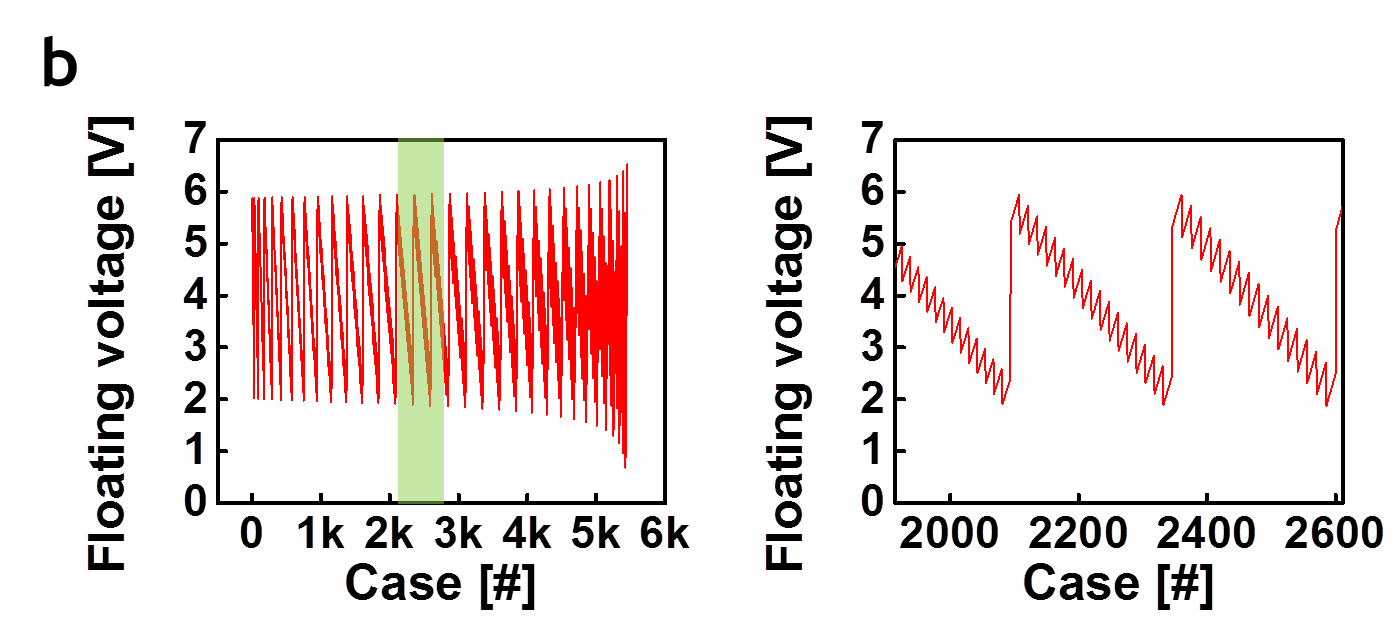
 Supplementary Figure S2 ǀ Floating voltage calculation of cross-point memristive synapse array. (a) Equivalent circuit for calculation of floating node voltage. (b) Floating node voltage plot for all possible cases.

For the worst case, it is assumed that a memristive synapse(memristor) has only two states, HRS(=10R, High resistance state) and LRS(=R, Low resistance state). For example, to update memristors’ state connected to first top electrode(TE), V_L_ and V_H_ are applied to each bottom electrode(BE) and V_CM_ is applied to fist TE. Also, second and third TE are floated. In this case, an equivalent circuit to calculate floating electrode(FE) can be presented as the right side of figure S3 (a). Where k1 and k2 are the number of LRS and HRS memristors with applied voltage V_H_, respectively. k3 and k4 are the number of LRS and HRS memristors with applied voltage V_L_, respectively. According to the Kirchhoff’s current low, I1 should be same as the summation of I2, I3 and I4. Thus, a floating voltage(F) could be determined with parameters such as k1, k2, k3, k4, N and k. Where N is the number of total memristor and k is the number of total HRS memristor. When N, V_H_ and V_L_ are 32, 2V and 6V, respectively, and all possible cases are considered, the plot of floating voltage is shown in figure S3 (b). It shows that the floating voltage varies from V_H_ to V_L_. Thus, unwanted weight changes of memristors are occurred when the voltage difference between FE and BE is larger than its threshold voltage. It is called as a unintended switching problem.


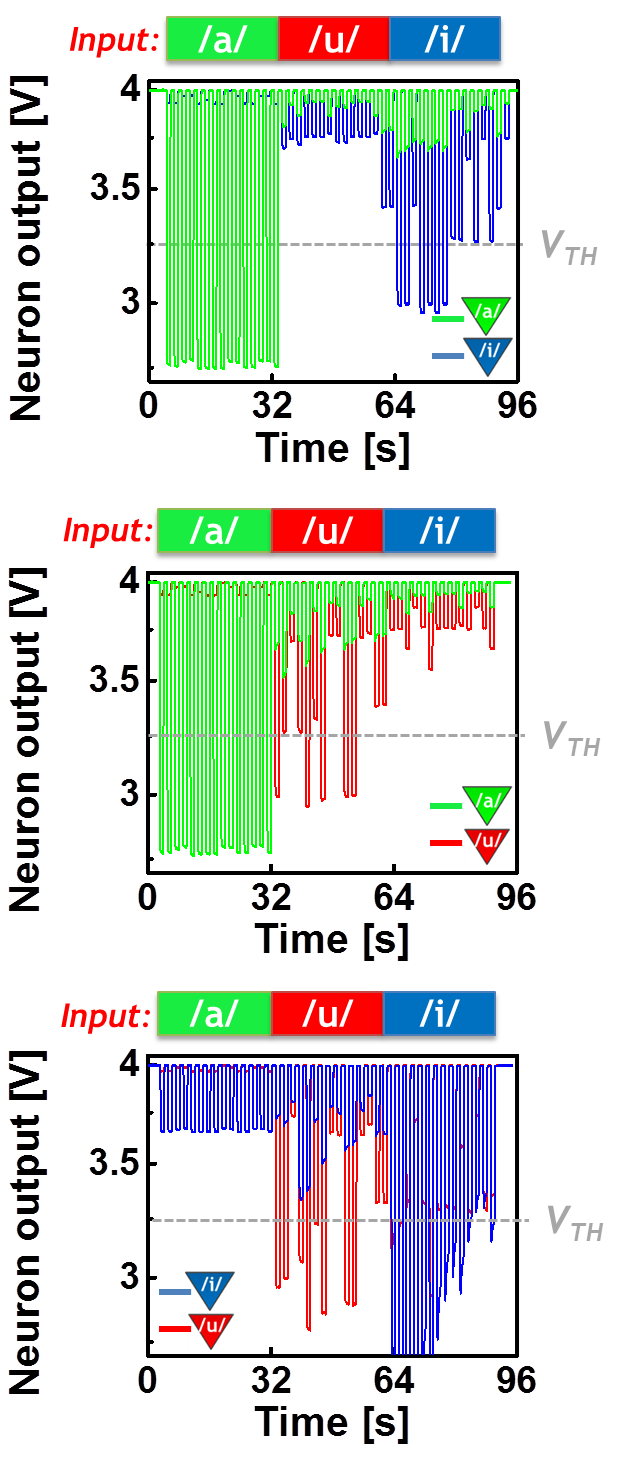


Supplementary Figure S3 ǀ Classification results for testing. The feature codes of /a/, /u/ and /i/ are sequentially applied to memristive HNN and 15 codes for each feature are used for test. The post-neurons are paired into 3 groups and each group compares two out of vowels, i. e. /a/ vs /i/, /a/ vs/u/ and /i/ vs/u/.

| **Feature Code** | | | | | | | | | | | | | | | | | | | | | | | | | | | | | | | | **Label Data** | **Speech** |
| --- | --- | --- | --- | --- | --- | --- | --- | --- | --- | --- | --- | --- | --- | --- | --- | --- | --- | --- | --- | --- | --- | --- | --- | --- | --- | --- | --- | --- | --- | --- | --- | --- | --- |
| **D30** | **D30** | **D29** | **D28** | **D27** | **D26** | **D25** | **D24** | **D23** | **D22** | **D21** | **D20** | **D19** | **D18** | **D17** | **D16** | **D15** | **D14** | **D13** | **D12** | **D11** | **D10** | **D9** | **D8** | **D7** | **D6** | **D5** | **D4** | **D3** | **D2** | **D1** | **D0** |
| 0 | 0 | 0 | 1 | 1 | 0 | 0 | 1 | 1 | 1 | 0 | 0 | 1 | 0 | 0 | 0 | 1 | 0 | 1 | 0 | 0 | 1 | 1 | 1 | 0 | 0 | 1 | 0 | 0 | 1 | 0 | 0 | 00 | /a/ |
| 0 | 0 | 0 | 1 | 1 | 1 | 1 | 0 | 0 | 1 | 1 | 0 | 1 | 1 | 1 | 0 | 0 | 0 | 1 | 1 | 0 | 1 | 1 | 0 | 1 | 0 | 1 | 0 | 0 | 0 | 0 | 0 | 00 | /a/ |
| 0 | 0 | 0 | 0 | 0 | 1 | 0 | 1 | 0 | 1 | 0 | 0 | 1 | 0 | 0 | 0 | 0 | 0 | 1 | 0 | 0 | 1 | 1 | 1 | 0 | 0 | 0 | 1 | 0 | 0 | 0 | 0 | 00 | /a/ |
| 0 | 0 | 0 | 1 | 0 | 0 | 1 | 0 | 0 | 1 | 1 | 0 | 1 | 0 | 0 | 0 | 0 | 0 | 1 | 1 | 0 | 0 | 1 | 0 | 0 | 0 | 1 | 0 | 0 | 1 | 0 | 0 | 00 | /a/ |
| 0 | 0 | 0 | 1 | 0 | 0 | 1 | 1 | 0 | 1 | 1 | 0 | 1 | 1 | 0 | 0 | 0 | 1 | 0 | 0 | 0 | 1 | 0 | 0 | 1 | 0 | 1 | 0 | 0 | 0 | 0 | 0 | 00 | /a/ |
| 0 | 0 | 0 | 1 | 0 | 0 | 0 | 0 | 1 | 0 | 0 | 0 | 0 | 0 | 0 | 0 | 0 | 0 | 1 | 1 | 0 | 0 | 1 | 0 | 1 | 0 | 0 | 0 | 1 | 0 | 0 | 0 | 00 | /a/ |
| 0 | 0 | 1 | 0 | 1 | 1 | 0 | 1 | 1 | 0 | 1 | 0 | 1 | 1 | 0 | 0 | 0 | 0 | 1 | 0 | 0 | 1 | 1 | 1 | 1 | 0 | 1 | 0 | 0 | 1 | 0 | 0 | 00 | /a/ |
| 0 | 0 | 0 | 0 | 1 | 0 | 1 | 0 | 1 | 0 | 1 | 0 | 0 | 0 | 1 | 0 | 0 | 0 | 0 | 0 | 0 | 0 | 1 | 1 | 0 | 0 | 1 | 0 | 0 | 0 | 0 | 0 | 00 | /a/ |
| 0 | 0 | 0 | 0 | 0 | 1 | 1 | 1 | 0 | 0 | 1 | 0 | 1 | 1 | 1 | 0 | 1 | 0 | 1 | 0 | 0 | 1 | 0 | 0 | 0 | 0 | 1 | 0 | 1 | 1 | 0 | 0 | 00 | /a/ |
| 0 | 0 | 0 | 1 | 0 | 1 | 1 | 1 | 0 | 1 | 0 | 0 | 1 | 1 | 0 | 0 | 0 | 1 | 1 | 0 | 0 | 1 | 1 | 0 | 1 | 0 | 1 | 0 | 0 | 1 | 0 | 0 | 00 | /a/ |

Supplementary Table S1 | An example of 32-bit feature code and 2-bit label data for speech /a/. In this work, the total number training and testing patterns are 65 and 15, respectively, they are different.

| **Feature Code** | | | | | | | | | | | | | | | | | | | | | | | | | | | | | | | | **Label Data** | **Speech** |
| --- | --- | --- | --- | --- | --- | --- | --- | --- | --- | --- | --- | --- | --- | --- | --- | --- | --- | --- | --- | --- | --- | --- | --- | --- | --- | --- | --- | --- | --- | --- | --- | --- | --- |
| **D30** | **D30** | **D29** | **D28** | **D27** | **D26** | **D25** | **D24** | **D23** | **D22** | **D21** | **D20** | **D19** | **D18** | **D17** | **D16** | **D15** | **D14** | **D13** | **D12** | **D11** | **D10** | **D9** | **D8** | **D7** | **D6** | **D5** | **D4** | **D3** | **D2** | **D1** | **D0** |
| 0 | 0 | 0 | 0 | 1 | 0 | 0 | 0 | 0 | 1 | 1 | 0 | 1 | 0 | 0 | 0 | 1 | 0 | 0 | 0 | 0 | 1 | 0 | 0 | 1 | 0 | 0 | 1 | 1 | 0 | 0 | 0 | 01 | /i/ |
| 0 | 0 | 0 | 0 | 1 | 0 | 0 | 1 | 0 | 1 | 0 | 0 | 0 | 1 | 0 | 0 | 0 | 1 | 1 | 1 | 0 | 1 | 0 | 0 | 0 | 0 | 0 | 1 | 0 | 0 | 0 | 0 | 01 | /i/ |
| 0 | 0 | 0 | 0 | 0 | 1 | 1 | 0 | 0 | 1 | 1 | 0 | 0 | 1 | 0 | 0 | 0 | 1 | 0 | 1 | 0 | 0 | 0 | 0 | 1 | 0 | 0 | 0 | 1 | 0 | 0 | 0 | 01 | /i/ |
| 0 | 0 | 0 | 0 | 0 | 1 | 0 | 0 | 1 | 0 | 1 | 0 | 1 | 0 | 0 | 0 | 0 | 1 | 0 | 1 | 0 | 0 | 1 | 1 | 0 | 0 | 0 | 0 | 1 | 1 | 0 | 0 | 01 | /i/ |
| 0 | 0 | 0 | 0 | 0 | 1 | 0 | 0 | 0 | 1 | 1 | 0 | 0 | 1 | 1 | 0 | 0 | 1 | 0 | 0 | 0 | 0 | 0 | 1 | 0 | 0 | 0 | 0 | 0 | 1 | 0 | 0 | 01 | /i/ |
| 0 | 0 | 0 | 0 | 0 | 0 | 1 | 0 | 0 | 1 | 0 | 0 | 0 | 1 | 1 | 0 | 0 | 1 | 1 | 0 | 0 | 0 | 0 | 1 | 1 | 0 | 0 | 0 | 0 | 1 | 0 | 0 | 01 | /i/ |
| 0 | 0 | 0 | 0 | 0 | 1 | 0 | 0 | 1 | 0 | 1 | 0 | 1 | 1 | 1 | 0 | 0 | 1 | 0 | 0 | 0 | 0 | 0 | 1 | 0 | 0 | 0 | 0 | 1 | 1 | 0 | 0 | 01 | /i/ |
| 0 | 0 | 0 | 0 | 0 | 1 | 1 | 0 | 0 | 0 | 1 | 0 | 1 | 0 | 1 | 0 | 0 | 1 | 0 | 1 | 0 | 0 | 0 | 1 | 0 | 0 | 0 | 0 | 0 | 1 | 0 | 0 | 01 | /i/ |
| 0 | 0 | 0 | 0 | 1 | 0 | 0 | 0 | 1 | 1 | 0 | 1 | 0 | 0 | 0 | 0 | 0 | 1 | 1 | 1 | 0 | 0 | 1 | 1 | 0 | 0 | 0 | 1 | 0 | 0 | 0 | 0 | 01 | /i/ |
| 0 | 0 | 0 | 0 | 0 | 1 | 0 | 0 | 1 | 0 | 0 | 0 | 1 | 0 | 1 | 0 | 0 | 1 | 0 | 1 | 1 | 0 | 0 | 0 | 0 | 0 | 0 | 1 | 1 | 0 | 0 | 0 | 01 | /i/ |

Supplementary Table S2 | An example of 32-bit feature code and 2-bit label data for speech /i/. In this work, the total number training and testing patterns are 65 and 15, respectively, they are different.

| **Feature Code** | | | | | | | | | | | | | | | | | | | | | | | | | | | | | | | | **Label Data** | **Speech** |
| --- | --- | --- | --- | --- | --- | --- | --- | --- | --- | --- | --- | --- | --- | --- | --- | --- | --- | --- | --- | --- | --- | --- | --- | --- | --- | --- | --- | --- | --- | --- | --- | --- | --- |
| **D30** | **D30** | **D29** | **D28** | **D27** | **D26** | **D25** | **D24** | **D23** | **D22** | **D21** | **D20** | **D19** | **D18** | **D17** | **D16** | **D15** | **D14** | **D13** | **D12** | **D11** | **D10** | **D9** | **D8** | **D7** | **D6** | **D5** | **D4** | **D3** | **D2** | **D1** | **D0** |
| 0 | 0 | 0 | 1 | 0 | 1 | 0 | 0 | 0 | 1 | 1 | 0 | 1 | 1 | 0 | 0 | 0 | 1 | 1 | 1 | 0 | 0 | 1 | 0 | 0 | 0 | 1 | 0 | 1 | 0 | 0 | 0 | 10 | /u/ |
| 0 | 0 | 0 | 0 | 0 | 1 | 1 | 0 | 1 | 0 | 0 | 0 | 0 | 0 | 1 | 0 | 1 | 1 | 0 | 0 | 0 | 0 | 0 | 1 | 0 | 0 | 0 | 1 | 0 | 1 | 0 | 0 | 10 | /u/ |
| 0 | 0 | 0 | 1 | 0 | 0 | 0 | 0 | 0 | 1 | 1 | 0 | 0 | 0 | 1 | 0 | 1 | 1 | 0 | 1 | 0 | 0 | 0 | 1 | 0 | 0 | 0 | 0 | 1 | 1 | 0 | 0 | 10 | /u/ |
| 0 | 0 | 0 | 1 | 0 | 1 | 1 | 1 | 1 | 0 | 0 | 0 | 0 | 1 | 1 | 0 | 0 | 1 | 0 | 0 | 0 | 0 | 1 | 0 | 1 | 0 | 1 | 1 | 0 | 0 | 0 | 0 | 10 | /u/ |
| 0 | 0 | 1 | 0 | 1 | 1 | 1 | 0 | 1 | 1 | 1 | 0 | 0 | 0 | 1 | 0 | 1 | 1 | 0 | 0 | 0 | 0 | 1 | 0 | 0 | 0 | 0 | 1 | 0 | 0 | 0 | 0 | 10 | /u/ |
| 0 | 0 | 0 | 1 | 0 | 1 | 0 | 0 | 1 | 1 | 0 | 0 | 0 | 1 | 1 | 0 | 1 | 0 | 0 | 1 | 0 | 0 | 0 | 1 | 1 | 0 | 0 | 1 | 0 | 1 | 0 | 0 | 10 | /u/ |
| 0 | 0 | 0 | 1 | 0 | 1 | 1 | 0 | 1 | 0 | 1 | 0 | 0 | 1 | 1 | 0 | 0 | 1 | 0 | 0 | 0 | 0 | 1 | 0 | 0 | 0 | 0 | 1 | 1 | 1 | 0 | 0 | 10 | /u/ |
| 0 | 0 | 0 | 1 | 1 | 0 | 0 | 0 | 1 | 1 | 0 | 0 | 1 | 0 | 1 | 0 | 1 | 1 | 0 | 0 | 0 | 0 | 1 | 0 | 0 | 0 | 0 | 1 | 0 | 0 | 0 | 0 | 10 | /u/ |
| 0 | 0 | 0 | 1 | 1 | 0 | 0 | 0 | 0 | 1 | 1 | 0 | 0 | 1 | 1 | 1 | 0 | 1 | 0 | 0 | 0 | 0 | 0 | 1 | 1 | 1 | 0 | 0 | 0 | 1 | 0 | 0 | 10 | /u/ |
| 0 | 0 | 0 | 1 | 0 | 1 | 1 | 0 | 0 | 1 | 0 | 1 | 0 | 0 | 0 | 0 | 1 | 1 | 1 | 0 | 0 | 0 | 0 | 1 | 0 | 0 | 0 | 1 | 1 | 1 | 0 | 0 | 10 | /u/ |

Supplementary Table S3 | An example of 32-bit feature code and 2-bit label data for speech /u/. In this work, the total number training and testing patterns are 65 and 15, respectively, they are different.

**
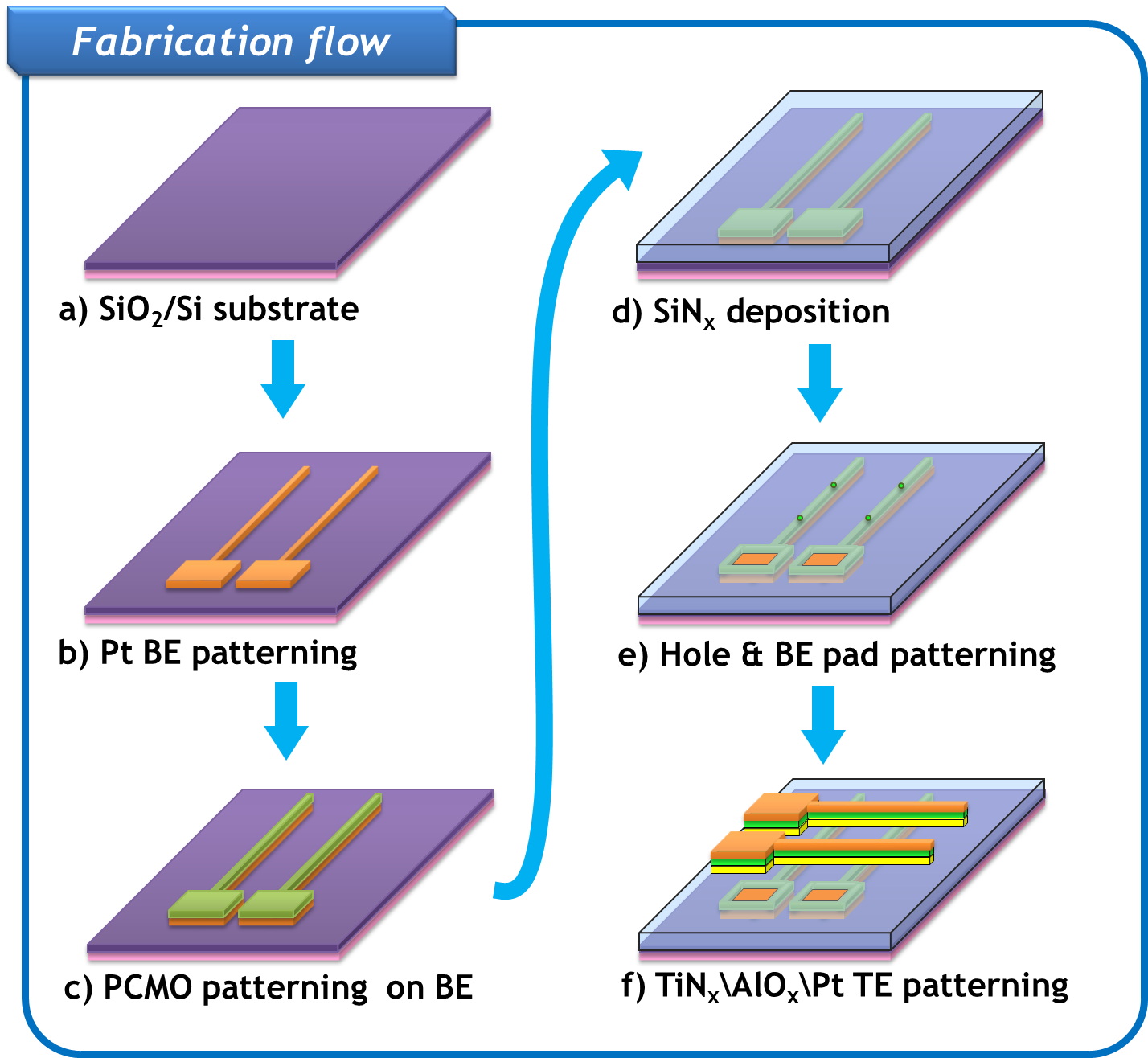
**

**Supplementary Figure S4 ǀ Fabrication process of memristive synapse.** (a) After initial 8 inch Si wafer cleaning, SiO_2_ oxide layer (300 nm) was deposited. (b) After plasma cleaning, Pt bottom electrodes (50 nm) were deposited by using evaporator and patterned by means of conventional lithography on SiO_2_/Si substrate. (c) Polycrystalline PCMO film (20 nm) was deposited by using RF magnetron sputter. During PCMO deposition, the substrate temperature was maintained at 600 ^o^C. Next, reactive ion etching was performed on Pt/SiO_2_/Si. (d) Next, a SiN_x_ layer (80 nm) was deposited by plasma-enhanced chemical vapor deposition. (e) In order to make via holes of the active area and BE pad, conventional lithography and reactive ion etching were performed. (f) The TE, which consisted of TiN_x_ (25 nm), AlOx (10 nm) and Pt (100 nm), was subsequently deposited (by RF magnetron sputtering) and patterned by conventional lithography.


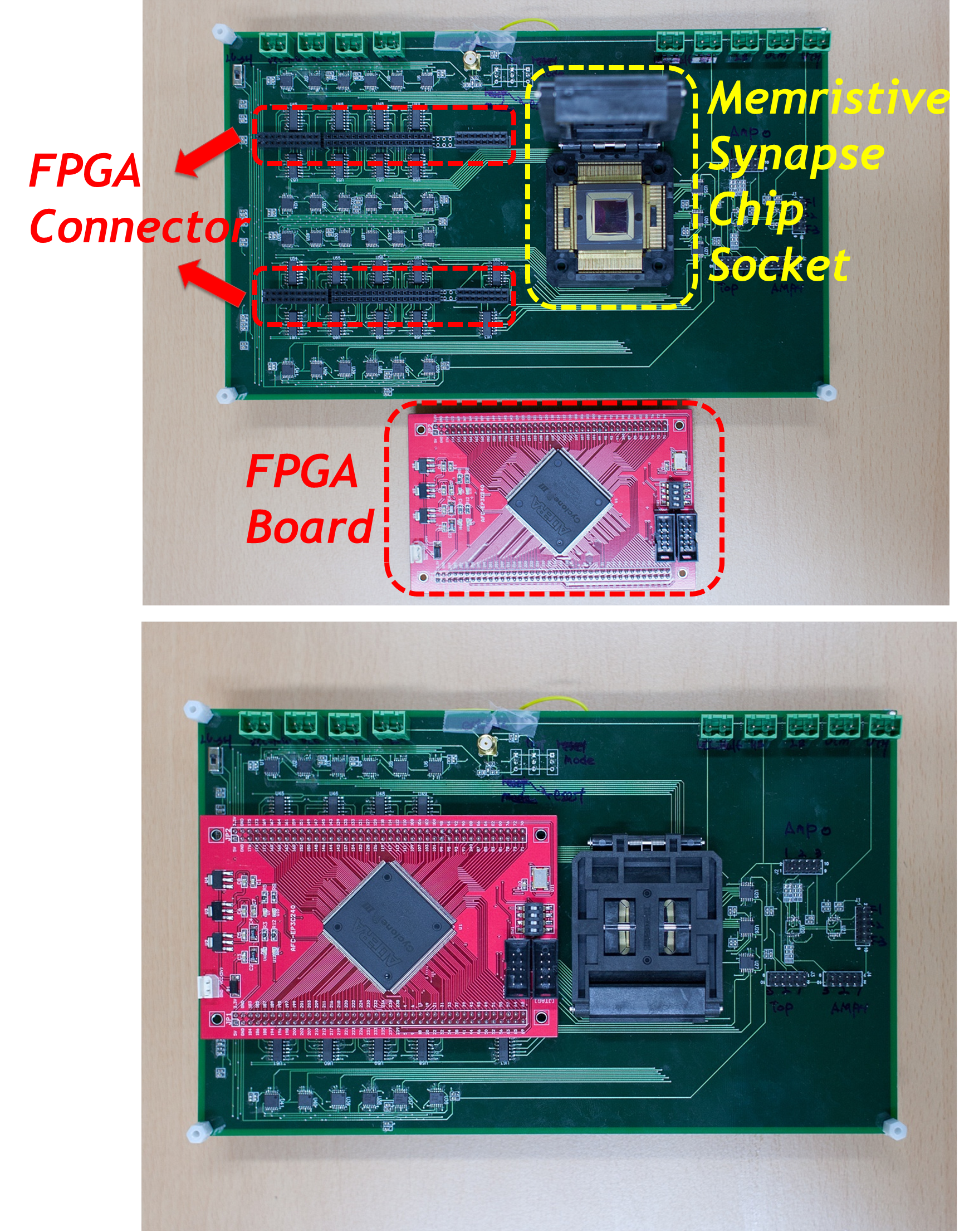


Supplementary Figure S5 ǀ Photo images of memristive hardware neural network(HNN). FPGA board is used as pre-neurons, controlling switch array to generate proper spike signals according to feature codes, and it is connected to main PCB board via FPGA connector. Also, cross-point memristive synapse array is packaged with quad flat package(QFP) and it is connected to main PCB via chip socket. Neurons circuits, including inverting integrator and comparator, are directly implemented on main PCB with commercial chip components
